# Development of an online food frequency questionnaire and estimation of misreporting of energy intake during the COVID-19 pandemic among young adults in Peru

**DOI:** 10.3389/fnut.2022.949330

**Published:** 2022-08-24

**Authors:** María Jesús Vega-Salas, Katherine Curi-Quinto, Alessandra Hidalgo-Aréstegui, Krysty Meza-Carbajal, Nataly Lago-Berrocal, Lena Arias, Marta Favara, Mary Penny, Alan Sánchez, Karani Santhanakrishnan Vimaleswaran

**Affiliations:** ^1^Carrera de Nutrición y Dietética, Departamento de Ciencias de la Salud, Facultad de Medicina, Pontificia Universidad Católica de Chile, Santiago, Chile; ^2^Hugh Sinclair Unit of Human Nutrition, Department of Food and Nutritional Sciences, University of Reading, Reading, United Kingdom; ^3^Instituto de Investigación Nutricional (IIN), Lima, Peru; ^4^Group for the Analysis of Development, Lima, Peru; ^5^World Food Programme of United Nations in Peru, Lima, Peru; ^6^Oxford Department of International Development, University of Oxford, Oxford, United Kingdom; ^7^The Institute for Food, Nutrition, and Health, University of Reading, Reading, United Kingdom

**Keywords:** food frequency questionnaire, dietary intakes, questionnaire validation, reliability, young adults, Latin American

## Abstract

**Background:**

The Young Lives longitudinal study switched to remote data collection methods including the adaptation of dietary intake assessment to online modes due to the physical contact restrictions imposed by the COVID-19 pandemic. This study aimed to describe the adaptation process and validation of an online quantitative food frequency questionnaire (FFQ) for Peruvian young adults.

**Methods:**

A previously validated face-to-face FFQ for the adult Peruvian population was adapted to be administered through an online self-administered questionnaire using a multi-stage process. Questionnaire development was informed by experts’ opinions and pilot surveys. FFQ validity was assessed by estimating misreporting of energy intake (EI) using the McCrory method, and the FFQ reliability with Cronbach alpha. Logistic regressions were used to examine associations of misreporting with sociodemographic, body mass index (BMI), and physical activity covariates.

**Results:**

The FFQ was completed by 426 Peruvian young adults from urban and rural areas, among whom 31% were classified as misreporters, with most of them (16.2%) overreporting daily EI. Men had a lower risk of under-reporting and a higher risk of over-reporting (OR = 0.28 and 1.89). Participants without a higher education degree had a lower risk of under-reporting and a higher risk of over-reporting (OR = 2.18 and 0.36, respectively). No major difference in misreporting was found across age groups, areas, studying as the main activity, being physically active or sedentary, or BMI. Results showed good internal reliability for the overall FFQ (Cronbach alpha = 0.82).

**Conclusion:**

Misreporting of EI was mostly explained by education level and sex across participants. Other sociodemographic characteristics, physical activity, sedentary behavior, and BMI did not explain the differences in EI misreporting. The adapted online FFQ proved to be reliable and valid for assessing dietary intakes among Peruvian young adults during the COVID pandemic. Further studies should aim at using and validating innovative dietary intake data collection methods, such as those described, for informing public health policies targeting malnutrition in different contexts after the COVID-19 pandemic.

## Introduction

An unhealthy diet is a major risk factor for non-communicable diseases (NCD), accounting for 11 million deaths (95% uncertainty interval [UI] 10–12) globally in 2017 (1). Measuring dietary intake is key for estimating the associations between diet and chronic diseases (2). There are several dietary assessment methods, including subjective reports and objective observation, open-ended surveys, such as dietary records, and closed-ended surveys, such as food frequency questionnaires (FFQs) (3). The latter is one of the most inexpensive and quickest methods for assessing usual food intakes over an extended period of time, and is extensively used by large-scale epidemiological studies (4). FFQs include a food list and a frequency response section, with the latter varying from open-or-closed-ended frequency responses, and optionally delimiting portion sizes (i.e., semiquantitative FFQ) (5). FFQs are appropriate when having limited resources for recording dietary intakes among a large number of participants (6), but they need to be developed and validated considering the different socioeconomic, cultural, and ethnic differences among the population targeted by the study (7). Even though an FFQ has been validated for the Peruvian population using face-to-face data collection methods (8), the FFQ used in our study has been further adapted to be administered as part of an online survey to estimate young people’s dietary intakes in an effective and efficient way during the COVID-19 pandemic.

One of the most prominent errors in dietary assessment is misreporting, which leads to implausible values for energy intakes (EI), occurring in around 30% of the respondents, regardless of the nutritional assessment method (9). Under-and over-reporting can be challenging as it can affect the direction and strength of associations between dietary intakes and health outcomes (10). Misreporting bias can be related to difficulties in recalling and averaging intakes over the long term, social desirability (e.g., reporting healthier foods and excluding unhealthy foods), and social environments characterized by a widespread weight bias (11, 12). Participants’ characteristics, including sex, age, and body weight, have also been associated with misreporting in studies conducted in different contexts (13–15). A study including Latin American countries (Argentina, Brazil, Chile, Colombia, Costa Rica, Ecuador, Peru, and Venezuela) identified sex, age, marital status, ethnicity, physical activity level (PAL), and Body Mass Index (BMI) nutritional status as key individual characteristics associated with misreporting of EI (16).

Peru is a middle-income country facing a double burden of malnutrition (DBM) characterized by the persistence of nutritional deficiencies, such as stunting and anemia, and a rapid increase in overweight and obesity rates among the adult population (37 and 21%, respectively) (17), but with dissimilar rates of under- and excess weight according to socioeconomic characteristics and geographical areas within the country (18, 19). During the COVID-19 pandemic, Peru became the country with the highest mortality rate worldwide by April 2022 (20). The lockdowns and reduced economic activity led to an increased food insecurity, especially among those from deprived backgrounds previous to the pandemic (21, 22). The rapid shifts in the DBM in Peru, with an increasingly overweight and obese adult population (23), makes it imperative to understand the dietary patterns of young adults by using a valid and reliable nutritional assessment method for this particular age group.

This study is a part of the Young Lives Study (YLS), known as “Niños del Milenio” in Peru, a longitudinal study tracking the livelihood of 12,000 children in four countries (Ethiopia, India (Andhra Pradesh and Telangana), Peru, and Vietnam) since 2002—a younger cohort born in 2001–2002, and an older cohort born in 1994–1995. In Peru, these cohorts were originally composed of 2,052 and 714 participants, respectively. In 2020, YLS planned to conduct the 6th visit to both cohorts when the subjects were aged 18–19 and 25–26 years (respectively). However, the COVID-19 pandemic imposed mobility restrictions and social distancing measures that made it impossible to collect data through face-to-face modes, and therefore, the YLS adapted its data collection mode to phone interviews and online self-administered questionnaires.

Hence, this study aims to describe the multi-stage process used to adapt a previously validated face-to-face FFQ of an online self-administered FFQ for young adults in Peru during the pandemic, including the selection of the food items, portion sizes, and food frequency response options. Furthermore, it aims at validating the FFQ by estimating the proportion and characteristics of misreporting of EI in this population and assessing the internal reliability of the online FFQ using Cronbach’s alpha test. Findings from this study are expected to inform the measurement of usual dietary intake in the YLS cohort in Peru. In addition, the tool designed can be a good alternative to develop nutritional studies with large coverage at a low cost in the context of developing countries.

## Materials and methods

### Study participants

**FIGURE 1:**
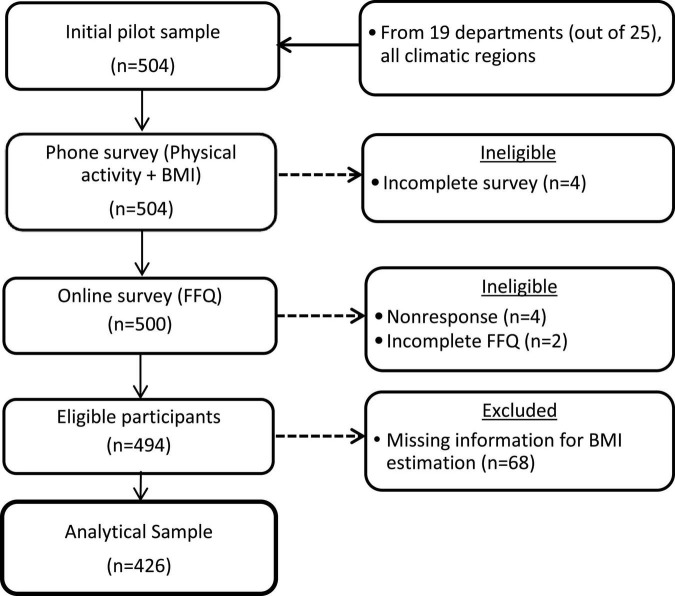
Flow chart showing the selection criteria for the identification of the analytical sample.

We analyzed data of 504 participants of the pilot study that was conducted in July 2021 to validate the online FFQ to be used as a dietary intake instrument for further YLS data collections. A convenience sample with similar characteristics as the ones from the YLS’s younger cohort (including the region of residence, aged 18–27 years, and a balanced distribution of sex) was invited to participate in the pilot study. For practical reasons, only participants with access to the internet and a computer or smartphone were invited to participate. Pregnant or breastfeeding women were excluded. Of the total sample, we excluded 78 participants for not responding or providing incomplete data either in the online FFQ and/or for height/weight measures, resulting in 426 participants (Figure 1). As part of the pilot study, participants were interviewed by phone and asked to self-report their weight and height, sociodemographic characteristics, and physical activity. After that, they were invited to complete an online survey *via* which the FFQ was administered.

### Development of the online food frequency questionnaire

**FIGURE 2:**
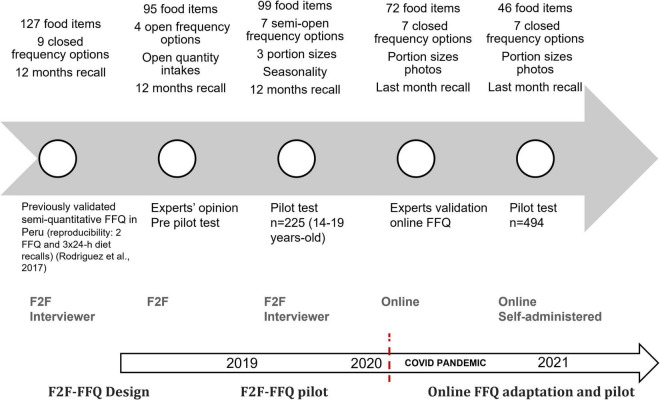
FFQ development processes. F2F, face-to-face; FFQ, food frequency questionnaire.

As is shown in Figure 2, the online FFQ was developed following a multi-stage process that included:

#### Designing a face-to-face food frequency questionnaire

We adapted a previously validated semi-quantitative FFQ that was developed to measure the dietary intake in children and adolescents in Lima, Peru (8). Briefly, this instrument was validated using three 24-h dietary recalls. The age-adjusted correlation coefficient between the FFQ and the mean of the 24-h dietary recalls was 0.33, a value considered as an acceptable validity (0.20–0.60) (24). This original FFQ was adapted for young adults based on the expert opinion of the YLS health researchers, a process led by a nutritionist specialized in the measurement of dietary intake from the Research Institute of Nutrition in Peru (IIN by its initials in Spanish in *Peru)*. Considering the aim of the YLS to estimate dietary EI using a comprehensive list of foods in a time-restricted period of application including another set of questions that could prompt the participant’s fatigue, the new adapted FFQ included the expert’s recommendation resulting in: **(i)** a reduced number of food items from 127 to 95 food items where the food items that are not commonly consumed by young adults were excluded as well as the food items with multiple presentations such as different kinds or brands of milk; **(ii)** delimiting nine closed frequency options (never, < 1 per month, 1 per week, 2–4 per week, 5–6 per week, 1 per day, 2–3 per day, and 4–5 per day) over the last 12 months to four open frequency options; and (**iii**) the addition of the option “quantity” and the inclusion of portion size photos for guiding the participants, which allow us to have a better approximation of the portions consumed by the study population.

#### Piloting the new face-to-face food frequency questionnaire version

The newly adapted version was piloted in a convenience sample of 225 participants aged 14–19 from two sites located in the southern part of the region of Lima (urban neighborhoods in the Cañete district located in the coastal area, and rural towns in the Yauyos province located in the highlands area). The face-to-face pilot study was conducted in 2019 to evaluate the participant’s comprehension and to observe the overall application process of the adapted FFQ. Trained YLS interviewers administered the adapted FFQ using the portion-size photos. The pilot study data was analyzed, and changes were included in the adapted version resulting in a new version with 99 food items, 7 semi-open frequency options (never, 1 per month, 2–3 per month, 1 per week, 2–4 per week, 5–6 per week, and daily), and a reference quantity for portion sizes and number of portions. The additional food items were excluded due to the low frequency of intake and different food presentations. For instance, around 92–99% have never consumed fresh skim milk, evaporated skim milk, “light” versions of products, such as sodas and yogurt, and hard cheese. In addition, the period for recalling food intake with the FFQ was reduced to the last month instead of the last 12 months because this facilitated the understanding and recall of the participants.

#### Adapting process from a face-to-face to an online food frequency questionnaire

As aforementioned, the COVID-19 pandemic made it impossible to conduct face-to-face fieldwork, so the YLS adapted its data collection methods for phone and online assessments; designing a new survey to measure the impact of the pandemic on both cohorts (25) and retaining the FFQ. Therefore, the adapted and piloted face-to-face FFQ was re-adapted for an online self-administered mode. This adaptation was performed by a panel of experts composed of two specialized nutritionists from the IIN, and a technical team to develop a digital software to assess dietary intake. To facilitate the comprehension and use of the self-administered online FFQ, it included specific instructions with examples at the beginning of the FFQ. In addition, questions about the frequency, portion sizes, and quantity of portion sizes were modified to be self-administered, and photos of the portion sizes for food items that are more difficult to identify were included. Furthermore, a help button that offers a simple explanation and examples was also included in the online FFQ.

The online self-administered FFQ was designed by a web developer who contributed to present a user-friendly FFQ layout that could be undertaken on small screens such as mobile phones. The online FFQ required access to the internet to fill it out. The final online FFQ was tested multiple times by the YLS research team of experienced interviewers to assure its functionality. Following this process, we reduced the number of food items from 72 to 46 to minimize the questionnaire self-application time considering that most of the participants would answer using mobile devices. We excluded food items based on the low frequency of consumption or merged them within the same group (e.g., Individual fruits were grouped according to the similarities in the nutritional content). We tested this new online version in the pilot study and the results are presented in this paper.

### Variables

#### Dietary assessment

We assessed the dietary intake by analyzing the data obtained from the online FFQ applied in the pilot study. We estimated the usual frequency of specific food intake during the last month and estimated the nutrient intake (energy, carbohydrate, protein, fat, iron, calcium, and fiber intake). The online FFQ was self-administered by each participant accessing the online platform. Participants were asked to recall the frequency and number of portions consumed during the last month, as well as the number of portions consumed on each occasion that they were eaten. For estimations of the quantity consumed per day, the number of days was multiplied by the number of times per day that the food item was consumed in the last month. For the estimation of the nutrient intake, a specific nutrient composition for each food item of the online FFQ was constructed based on the food composition database built with data from the *IIN, Lima-Peru*, which has information from local foods; the Peruvian food composition table of the National Center for Food and Nutrition of Peru (CENAN) and a Latin-American food composition table.

#### Body mass index

Body mass index (BMI) was used as an indicator of nutritional status that is calculated by dividing weight in kilograms by the square of height in meters (kg/m^2^). The body weight and height were reported in the phone survey used in the pilot study of the online FFQ. The BMI was classified as overweight (BMI ≥ 25 and < 30 kg/m^2^), obesity (BMI ≥ 30 kg/m^2^), and underweight (BMI < 18.5 kg/m^2^) (26).

#### Sociodemographic characteristics

Prior to the online survey, through the phone survey, we collected sociodemographic characteristics of each respondent, including sex, age (18–22 and 23–27 years old), geographical area (urban and rural), education level (secondary education or lower and higher than secondary education), and main activity (studying or working).

### Validation of the online food frequency questionnaire using the misreporting of energy intake

We estimated the misreporting of EI as a way to validate the online FFQ. Misreporters were identified using the method developed by McCrory et al. (27). One of the most common procedures used for identifying inaccurate reports of EI is the method that was first developed by Goldberg et al. (28), which identifies under-reporters (U-R), over-reporters (O-R), and plausible reporters (P-R) of EI. This method estimates a confidence interval (CI) for the level of agreement between PAL and the ratio of EI to Basal Metabolic Rate (BMR) based on the coefficients of variation (*CV*) of subjects’ EI ($C\,V_{w\,E\,I}^{2}$), BMR ($C\,V_{w\,B}^{2}$), and PAL ($C\,V_{t\,P}^{2}$).

Since this first approach, many other procedures have been developed to identify misreporting based on the comparison between EI and Estimated Energy Requirement (EER) using CI and cut-off points. In an attempt to overcome some of the problems associated with the most classical methods, McCrory et al. (27) developed an alternative approach to the one originally proposed by Goldberg et al. (28). The use of this method will allow us to theoretically eliminate the potential error of assigning inaccurate PALs with only limited information on the activity of individuals under study (27). From the below equation, the SD is calculated from the $C\,V_{w\,E\,I}^{2}$ over the numbers of days of diet assessment (*d*), $C\,V_{w\,p\,T\,E\,E}^{2}$ is the CV measuring the total energy expenditure (TEE) by the doubly labeled water method and $C\,V_{p\,T\,E\,E}^{2}$ is the CV for predicting the TEE.


$$\pm S\,D=\sqrt{\frac{C\,V_{w\,E\,I}^{2}}{d}+C\,V_{w\,p\,T\,E\,E}^{2}+C\,V_{p\,T\,E\,E}^{2}}$$

According to the results of the Latin American Study of Nutrition and Health (ELANS) study for the Peruvian sample, the $C\,V_{w\,E\,I}^{2}$ is set to 26.18% and based on two 24-h dietary recall (*d*) (16). $C\,V_{w\,p\,T\,E\,E}^{2}$ use is 8.2% from the estimations of Black (29) and $C\,V_{p\,T\,E\,E}^{2}$ 31.2% according to results of Vinken et al. (30). From the above formula, it is possible to calculate the ± 2 SD for the agreement between of the reported EI and the predicting TEE.

### Assessment of reliability of the online food frequency questionnaire

The reliability of the FFQ was assessed by estimating the internal consistency reliability of the overall FFQ, which included 14 different food groups, and within food groups. For this purpose, we used Cronbach’s alpha, a widely used statistic for estimating the internal consistency of items within a scale (31, 32). Previous studies have used Cronbach’s alpha for estimating the FFQ internal consistency reliability (33, 34).

### Ethics

The YLS in Peru has been approved by the ethical board of the *Instituto de Investigación Nutricional*, IIN (180-2002/CEI-IIN) and by the Oxford Department of International Development (SSD/CUREC2/07-026). Subsequently, each new survey round has received approval from these departments. All participants were informed in each of the data collection phases and gave consent to participate in the study. Approval for the data under analysis was obtained from the IIN Ethical Board in June 2021 (157-2021/CIEI-IIN).

### Statistical analysis

For discrete variables, a Chi-squared test is applied to evidence differences among the U-R and O-R groups in the characteristics of the participants (Table 1). Similarly, the Kruskal-Wallis illustrated differences among misreporting groups for the total energy and nutrient intake (Tables 2, 3). Logistic regressions reporting odds ratio (OR), *p*-value, and 95% confidence intervals (CI) were used to assess the risk of being classified as an over-reporter, compared to a plausible reporter, according to the McCrory method (Table 4). All analyses were carried out using the STATA software (version 16.1; College Station, TX: StataCorp LLC). *p* < 0.05 was considered statistically significant.

**TABLE 1:** Characteristics of the participants stratified by misreporting status.

	All sample		Plausible reporters (P-R)	Under-reporters (U-R)	Over-reporters (O-R)	Chi2 *P*-value	
			
	Mean/% (*N*)	*SD*	Mean/%	Mean/%	Mean/%	U-R	O-R
Total	100 (426)	−	69.0	14.8	16.2	−	−
**Sex**							
Men	50.2 (214)	0.4	72.0	7.5	20.6	0.000	0.014
Women	49.7 (212)	0.3	66.0	22.2	11.8		
**Age group**							
18–22 years-old	59.9 (255)	0.4	68.2	12.9	18.8	0.190	0.072
23–27 years-old	40.1 (171)	0.4	70.2	17.5	12.3		
**Area of residence**							
Rural	12.0 (51)	0.3	64.7	21.6	13.7	0.146	0.610
Urban	88.0 (373)	0.4	69.6	13.9	16.5		
**Climatic region**							
Coast	42.72 (182)	0.4	70.3	14.3	15.4	0.839	0.050
Highlands	33.3 (142)	0.3	71.8	16.2	12.0		
Jungle	23.9 (102)	0.4	62.7	13.7	23.5		
**Education**							
Secondary education or lower	77.2 (97)	0.4	69.9	11.6	18.5	0.001	0.016
Higher than secondary education	22.8 (329)	0.3	66.0	25.8	8.2		
**Studying as a primary activity**							
No	32.9 (140)	0.4	62.9	19.3	17.9	0.067	0.515
Yes	67.1 (286)	0.4	72.0	12.6	15.4		
**Nutritional status (BMI category)**							
Underweight	3.3 (14)	0.4	71.4	14.3	14.3		
Normal	60.8 (259)	0.4	68.3	15.8	15.8	0.887	0.969
Overweight	29.8 (127)	0.4	70.1	13.4	16.5		
Obese	6.1 (26)	0.5	69.2	11.5	19.2		
**Sedentary**							
No	66.2 (282)	0.4	67.7	14.9	17.4	0.932	0.355
Yes	33.8 (144)	0.4	71.5	14.6	13.9		
**Active**							
No	74.2 (316)	0.4	70.9	13.9	15.2	0.394	0.339
Yes	25.8 (110)	0.4	63.6	17.3	19.1		

Higher than secondary education includes university (undergraduate and postgraduate studies) as well as non-university education (vocational and technical training); Sedentary: spends more than 4 h in a seated position; Active: 4 days or more a week doing at least 1 h of active exercise. P-R: Plausible reporters; U-R: Under reporters; O-R: Over reporters; SD: Standard deviation; BMI: Body mass index. The significance among categories was assessed using the Chi-square test for two mean comparisons of proportions and ANOVA with the Bonferroni multiple comparison test for comparison of more than two categories.

**TABLE 2:** Total energy intake and nutrient intake by misreporting status.

	All sample		Plausible reporters (P-R)		Under-reporters (U-R)		Over-reporters (O-R)		Kruskal-wallis H test	
					
	Mean (*SD*)	Median (P25-P75)	Mean (*SD*)	Median (P25-P75)	Mean (*SD*)	Median (P25P75)	Mean (*SD*)	Median (P25–P75)	PR vs. U-R	PR vs. O-R
Total energy intake (EI)	4445.3 (3300.6)	3596.3 (2452.5–4970.2)	3672.6 (1088.8)	3541.3 (2708.4–4339.6)	1631.8 (295.0)	1712.9 (1443.3–1863.4)	10306.6 (4243.3)	9051.3 (8045.8–10236.4))	0.00	0.00
Carbohydrate (% EI)	59.0 (9.7)	59.1 (52.6–65.6)	59.2 (9.2)	59.3 (53.2–65.4)	60.7 (10.1)	60.0 (55.9–68.9)	56.6 (11.0)	57.7 (48.6–63.6)	0.00	0.00
Carbohydrate (g)	646.8 (491.6)	520.1 (358.1–756.3)	541.3 (175.6)	516.3 (406.1–655.7)	248.4 (63.6)	253.4 (196.1–288.2)	1460.2 (709.4)	1287.0 (1031.6–1511.2)	0.00	0.00
Protein (% EI)	17.5 (3.5)	17.2 (15.1–19.5)	17.5 (3.4)	17.3 (15.1–19.4)	18.4 (3.8)	18.4 (15.6–20.8)	16.8 (3.6)	16.4 (14.9–18.3)	0.00	0.00
Protein (g)	192.3 (149.2)	154.1 (105.9–220.9)	160.4 (56.5)	153.4 (114.2–189.8)	74.3 (17.4)	73.6 (62.0–84.0)	436.0 (217.2)	382.1 (326.1–437.0)	0.00	0.00
Total fat (% EI)	24.3 (6.4)	23.9 (19.7–28.2)	24.1 (5.9)	23.9 (20.0–28.0)	22.9 (6.7)	22.3 (17.3–26.4)	26.3 (7.6)	25.7 (21.4–31.7)	0.00	0.00
Total fat (g)	123.4 (106.4)	90.9 (62.7–145.9)	99.1 (41.1)	90.5 (70.4–116.8)	41.5 (14.0)	42.9 (29.6–50.9)	302.1 (148.1)	263.2 (207.6–342.2)	0.00	0.00
Iron (mg)	36.7 (30.2)	29.2 (20.4–41.9)	30.8 (10.7)	29.1 (23.4–36.7)	14.6 (3.1)	14.8 (11.5–17.1)	81.6 (50.3)	67.2 (58.0–80.7)	0.00	0.00
Calcium (mg)	1288.9 (1301.2)	940.1 (618.2–1450.9)	1036.4 (481.8)	935.9 (696.5–1248.9)	437.9 (160.5)	410.8 (323.4–530.8)	3141.8 (2253.0)	2407.8 (1834.4–3513.7)	0.00	0.00
Fiber (g)	21.6 (20.4)	15.8 (11.1–25.2)	17.3 (7.7)	15.7 (12.2–21.8)	8.0 (3.5)	7.5 (5.9–9.6)	52.2 (33.6)	41.6 (32.7–56.9)	0.00	0.00

EI, Energy Intake; g, grams; mg, milligrams; SD, Standard deviation; P-R, Plausible reporters; U-R, Under reporters; O-R, Over reporters.

**TABLE 3:** Food groups contribution to total energy intake by misreporting status.

	All sample		Plausible reporters (P-R)		Under-reporters (U-R)		Over-reporters (O-R)		Kruskal-wallis H test	
					
	Mean (*SD*)	Median (P25-P75)	Mean (*SD*)	Median (P25-P75)	Mean (*SD*)	Median (P25-P75)	Mean (*SD*)	Median (P25-P75)	PR vs. U-R	PR vs. O-R
Group 1: Cereals	21.6 (11.5)	19.1 (13.5–28.9)	22.0 (11.2)	19.4 (13.9–28.9)	26.5 (13.1)	26.3 (15.9–34.9)	15.4 (8.3)	13.9 (9.1–19.6)	0.00	0.00
Group 2: Whole grains	1.5 (2.7)	0.6 (0.2–1.6)	1.6 (3.0)	0.6 (0.2–1.6)	1.0 (1.2)	0.5 (0.2–1.3)	1.4 (2.6)	0.8 (0.2–1.7)	0.27	0.71
Group 3: Starchy	14.2 (9.3)	12.5 (7.2–18.8)	14.3 (9.0)	13.1 (7.2–19.7)	14.6 (10.9)	11.1 (7.5–20.8)	13.6 (9.1)	11.0 (7.2–17.4)	0.52	0.18
Group 4: Stew/menestras (vegetables)	4.9 (5.1)	3.3 (1.6–5.9)	5.0 (5.1)	3.4 (1.6–6.2)	5.1 (4.6)	3.6 (1.7–8.1)	4.3 (5.6)	2.9 (1.4–5.0)	0.34	0.01
Group 5: Nuts and seeds	0.7 (1.8)	0.2 (0.0–0.6)	0.7 (1.9)	0.2 (0.0–0.5)	0.7 (1.2)	0.3 (0.0–0.9)	0.7 (1.5)	0.2 (0.0–0.4)	0.23	0.79
Group 6: Dairy	5.5 (4.8)	4.3 (2.2–7.3)	5.6 (4.6)	4.3 (2.4–7.4)	4.8 (4.2)	4.0 (1.6–7.1)	6.0 (6.2)	4.1 (2.0–8.3)	0.00	0.95
Group 7: Animal protein	13.9 (7.4)	12.7 (8.7–17.4)	13.9 (7.3)	12.8 (8.3–18.0)	13.7 (8.2)	12.3 (8.5–17.2)	13.6 (6.8)	12.6 (10.1–17.0)	0.26	0.73
Group 8: Seafood	2.6 (2.6)	1.8 (1.0–3.3)	2.4 (2.3)	1.6 (0.9–3.0)	3.0 (2.9)	2.1 (1.2–3.6)	3.0 (3.0)	2.0 (1.0–4.1)	0.01	0.11
Group 9: Vegetables	2.8 (3.4)	1.9 (1.0–3.6)	2.6 (2.4)	1.8 (1.0–3.3)	3.3 (3.3)	2.8 (1.1–4.7)	3.4 (5.9)	1.9 (1.0–3.6)	0.00	0.44
Group 10: Fruits	8.0 (5.9)	6.7 (3.7–10.3)	7.7 (5.4)	6.5 (3.5–10.1)	8.0 (6.2)	6.6 (3.8–10.0)	9.5 (7.4)	7.0 (4.4–12.5)	0.57	0.00
Group 11: Refined cereals	5.1 (4.7)	3.9 (1.9–6.8)	4.7 (4.0)	3.8 (1.8–6.5)	4.1 (3.2)	3.2 (1.8–5.8)	7.7 (7.1)	5.3 (2.7–11.8)	0.00	0.00
Group 12: Added fats	10.7 (7.2)	9.3 (5.0–14.8)	10.8 (6.9)	9.5 (5.4–14.9)	8.9 (7.5)	6.1 (3.4–11.5)	11.9 (7.8)	10.6 (5.8–16.2)	0.00	0.00
Group 13: Added sugars	6.6 (5.5)	5.4 (2.7–8.8)	6.9 (5.3)	5.8 (3.2–9.0)	5.4 (4.4)	4.3 (2.1–8.0)	6.4 (6.8)	4.7 (2.1–9.0)	0.00	0.00

SD, Standard deviation; P-R, Plausible reporters; U-R, Under reporters; O-R, Over reporters.

**TABLE 4:** Association of sociodemographic factors with misreporting status.

Socio demographic factors	Under-reporters (U-R)			Over-reporters (O-R)		
		
	Odds ratio	95% CI	*P* > | z|	Odds ratio	95% CI	*P* > | z|
Sex (ref. Women)						
Men	0.28	[0.13; 0.62]	0.00	1.89	[1.09; 3.28]	0.02
	(0.11)			(0.53)		
Age group (ref. Younger Cohort)						
Older cohort (23–27)	1.15	[0.65; 2.05]	0.63	0.62	[0.34; 1.15]	0.13
	(0.34)			(0.20)		
Residence area (ref. Rural)						
Urban	0.62	[0.31; 1.25]	0.18	1.18	[0.66; 2.14]	0.58
	(0.22)			(0.36)		
Educational level (ref. Secondary education or lower)						
Higher than secondary education	2.18	[0.94; 5.07]	0.07	0.36	[0.18; 0.70]	0.00
	(0.94)			(0.12)		
Studying status (ref. Not studying)						
Study: Yes	0.80	[0.41; 1.55]	0.51	0.54	[0.26; 1.13]	0.10
	(0.27)			(0.20)		
Nutritional status (BMI category) (ref. Normal weight)						
Underweight	0.84	[0.17; 4.18]	0.84	1.03	[0.24; 4.49]	0.97
	(0.69)			(0.77)		
Overweight	0.84	[0.47; 1.48]	0.54	1.14	[0.61; 2.13]	0.67
	(0.24)			(0.36)		
Obese	0.61	[0.13; 2.85]	0.53	1.44	[0.46; 4.52]	0.53
	(0.48)			(0.84)		

Robust standard errors in parentheses. OR U-R, U-R vs. PR+O-R; OR O-R, O-R vs. PR+U-R. P-R, Plausible reporters; U-R, Under reporters; O-R, Over reporters; CI, Confidence intervals; ref., Reference category.

Internal consistency reliability of the online FFQ was assessed with Cronbach’s alpha. Before conducting the analysis, the food intake values were log-normalized to avoid any possible disturbance due to non-normal distributions (35, 36).

## Results

The pilot test that was conducted in 2021 *via* phone and online modes included 426 participants (analytical sample) from 19 different departments and from the three climatic regions in Peru (Table 1). About 69% of the participants were classified as P-R, 16.2% as O-R, and the remaining as U-R. The sample was sex- and age-group balanced, representing nearly 50% for each category. Among men, 72% of them were classified as P-R, while this share scales up to 66% among women. In addition, 21% of the men were identified as O-R and 22% of the women as U-R. Furthermore, 68% of participants aged 18–22 years old were classified as P-R, and 19% as O-R, whereas among 23–27 years old, 18% were classified as U-R. Regarding the area of residence, 88% of the participants lived in urban areas, and 70 and 65% of those living in urban and rural areas were classified as P-R, respectively. Likewise, 43, 33, and 24% of participants lived in the coast, highlands, and jungle regions, respectively, with 70, 72, and 63% of participants from each region assigned as P-R, respectively. The jungle region concentrated a higher proportion of O-R (24%). With respect to education, only 23% of the participants have completed a higher education (HE) degree (vocational or university), among those, 66% are classified as P-R, yet 26% were classified as U-R, whereas 19% of those from the lower education level were identified as O-R. Furthermore, 67% were studying as a primary activity, and 72% were classified as P-R. Regarding the participant’s nutritional status and physical activity, 61% were classified as normal weight, while 30% were classified as overweight, and 6% as with obesity. No major differences among P-R were found according to the nutritional status categories, reaching around 70% among all the participants. Small differences suggesting a lower U-R and higher O-R among participants with obesity were identified, when compared to their normal weight counterparts. Additionally, 34% of the participants were sedentary, 74% were non-active, and 72 and 64% were identified as P-R, respectively.

Misreporting of EI estimation was conducted by using the McCrory method, with a prevalence of 14.8% of U-R and 16.2% of O-R, respectively. Logistic regressions show the odds ratio (OR) for the risk of being a U-R and O-R compared with a P-R, according to sociodemographic characteristics and nutritional status (Table 4). On the one hand, results show that men have a 72% lower risk of being a U-R, when compared to women. On the other hand, men have an 89% higher risk of being an O-R, when compared to women. Additionally, having a HE degree significantly increases the risks of being an O-R by 1.18 times, when compared to participants with a lower educational level. Furthermore, having a HE degree reduces the risk of being an O-R by 64%, when compared to participants with a lower educational level. Neither the age group (19–22 vs. 23–27), living in urban vs. rural areas, studying as a primary activity nor the nutritional status when contrasted to the normal weight category presented a significant difference in U-R nor O-R.

As shown in Table 2, P-R presented a mean and median total EI of 3,673 and 3,541 kCal/day, respectively, which is significantly higher than 1,632 and 1,713 kCal/day among U-R, and lower than 10,307 and 9,051 kCal/day for O-R. Regarding the relationship between macronutrients and EI, U-R has a relatively higher % of total EI from carbohydrates and protein, whereas O-R has a relatively higher % for total fat intake. When comparing the food group’s contribution to total EI to P-R, U-R presented a significantly higher contribution from cereals and vegetables, and a lower relative contribution from dairy products, refined cereals, added fats, and added sugars (Table 3). Meanwhile, O-R showed a relatively higher contribution from fruits, refined cereals, added fats, and added sugars, and a lower contribution from cereals, stews and added sugars, when compared to P-R. Overall, the piloted FFQ shows good internal consistency with a Cronbach’s alpha of 0.82 for food groups (Supplementary Table 1), which is higher than the recommended 0.7 threshold (31, 32). The exclusion of individual food groups varies the total Cronbach’s alpha from 0.801 (refined cereals) to 0.819 (whole grains), supporting the FFQ reliability for measuring dietary intakes. Three food groups (cereals, starchy, and stews) present insufficient internal consistency with Cronbach’s alpha values ranging from 0.26 to 0.54. Exclusion of individual items was found to improve their food group Cronbach’s alpha, mainly due to its low-frequency intake (Supplementary Table 2).

## Discussion

Our study aims at adapting, developing, and validating a self-assessed online FFQ administered during the COVID pandemic to younger adults (18–27 years old) in Peru. It describes a detailed account of the multi-stage process for adapting a previously validated face-to-face FFQ to an online self-administered dietary intake questionnaire. Together with the analysis of the misreporting of EI and its associated characteristics, we have assessed the reliability by measuring the internal consistency of the FFQ and its food groups. While previous studies have assessed misreporting of EI within Latin-American countries (16), to our knowledge, this is the first study assessing misreporting of EI and its related characteristics together with estimating the internal consistency of a self-administered online FFQ.

Our study (*n* = 426) reported that 14.8 and 16.2% of the participants can be classified as U-R and O-R, respectively, with mean total EI values of 1,632 and 10,307 kCal/day (SD U-R = 295; 4,243). Our results were similar to those from the ELANS study including adults from eight Latin-American countries (*N* = 9,218), which reported 12.1% U-R and 14.1% O-R with means for total EI of 5,570 and 11,567 kCal, respectively (*SD* = 1,429 and 2,828, respectively). A study including US adults based on data from NHANES 2003–2012 (*N* = 19,396) reported 25.1% U-R and only 1.4% O-R (37). While a study in the US children and adolescents aged 2–19 years (*N* = 14,044) reported 13.1% U-R and 5.4% O-R (38), and a study in post-pubertal Brazilian adolescents (*N* = 96; mean age = 16.6) reported 64.6% U-R and 1% O-R (39), an Australian study from the Childhood Determinants of Adult Health Study (CDAH) including adults aged 26–36 years-old (*N* = 1,919) reported 28.6% U-R and 6.1% O-R (40). The larger variation in the prevalence of misreporting in comparison with previous studies from diverse contexts can be attributed to differences in the dietary intake assessment methods and cutoff values for delimiting misreporting used by different studies. Our study assessed dietary intake with a semi-quantitative FFQ and estimated misreporting by using the McCrory method (± 2 SD). The ELANS study assessed two non-consecutive 24-h dietary recalls (24HR) and used the McCrory method with a cutoff value of ± 1.5 SD based on pTEE (16). Moreover, the NHANES study used two 24-h dietary recall for each of the seven rounds included in the analysis and used the Goldberg method with a cutoff value of ± 2 SD of the ratio of EI to BMR [Murakami and Livingstone (37)]. The Australian CDAH study assessed EI using a qualitative FFQ and estimated misreporting by using the Goldberg method (± 2 SD) and the predicted total energy expenditure method (± 1.5 *SD*) (40).

Higher misreporting of EI in our study is associated with sex and education, with women and higher educated participants at higher risk of U-R, and men and low-educated participants for O-R. These findings are consistent with previous studies in Latin American countries (16). In contrast, a study in the US adults presented higher risks of O-R among men and underweight participants, whereas older adults, low-educated participants with lower family poverty income ratio, and participants with overweight and obesity were at higher risk for both, U-R and O-R (37). Meanwhile, among US children and adolescents, the risk of being O-R was related to a lower family poverty income ratio and younger age children (2–5 years old) (38). Our findings suggesting a higher risk of U-R among women and O-R among men are similar to several studies, thereby providing evidence for the association between misreporting and sex, regardless of their age and other characteristics (16, 37, 41, 42). The reasons underpinning these sex differences can relate to the higher social desirability regarding EI among women, who tend to underreport more to match dietary guidelines and media messages regarding healthy eating (13). Additionally, women across the globe encounter different sociocultural pressures toward thin and slender body ideals that are imposed by western media (43). These body ideals are introduced from developed to developing countries by colonial and social class differentiation processes that are based on racial and body type ideals that do not necessarily reflect the perceived ideals for Peruvian or Latin American women (44, 45). As obesity rates among women from developed and developing countries are increasing at a speedy rate (46, 47), matching the thin body ideals becomes an impossible goal to achieve (48). Therefore, the social desirability of thinness may contribute to the underreporting of EI among women found in our study. In contrast, the reasons behind the relatively higher risk of men for O-R are not clear and require further investigation.

Our results have underpinned a significant negative association between education level and misreporting, consistent with several studies suggesting a higher risk of U-R among the higher educated, and higher O-R among the low-educated participants (16, 37). However, other associated variables assessing socioeconomic position (SEP), including education, income, and occupation (49, 50), have also been related to misreporting (41). For example, the ELANS study reported a higher risk of U-R among the low-educated participants but a higher O-R among those from the lower SEP (16). The NHANES study reported a higher risk of O-R among the higher family income-poverty ratio compared to children and adults from the lower family income-poverty ratio (37, 38). On the one hand, one of the main explanations given by the literature for the higher risk of misreporting among those from the lower SEP is the relative poorer literacy skills among this group, which could impact the questionnaire’s comprehension and the relative higher social desirability due to a higher health or diet consciousness leading to under-reporting or report plausible values among better-off socioeconomic groups (41). Despite the fact most of the participants in our study had completed secondary education, international standardized tests have concluded that Peruvians aged 25–65 years with secondary education or below have the lowest scores for reading comprehension and mathematics among a group of Latin American countries (51). Therefore, this underperformance in key competences among the lowest educated could have impacted the FFQ comprehension. Moreover, education has also been related to social desirability of self-reported dietary intakes, with the higher educated under-reporting and reporting plausible EI more than their lower educated counterparts (13).

Previous studies including adults and children from different regions have highlighted the association between misreporting of EI and specific anthropometric and socio-demographic characteristics, including high BMI, being a woman, and having a low education level (13, 42). Our findings did not encounter significant differences between nutritional status nor physical activity or sedentary behaviors and the risk of misreporting, yet several studies have reported differences in misreporting according to these characteristics. Latin American individuals with overweight and obesity have been identified as having a greater risk of U-R, whereas underweight of O-R (16). The underlying reasoning for misreporting among the more extreme BMI categories can be related to the stigma these populations experience (52). As such, individuals deviant from the normal weight category can have their body image affected and be more prone to respond to what is considered to be socially desirable (13, 53). Other sources of misreporting of EI can be due to unconscious incomplete recordkeeping (e.g., due to omission of eating occasion/item, memory fatigue, portion size misrepresentation) or conscious misreporting (e.g., due to social desirability) (13). Despite all nutritional surveys being prone to a certain degree of misreporting (16), identifying the characteristics related to this phenomenon can offer insights for developing mitigation strategies to minimize the systematic bias among the participants of similar age groups and contexts.

The second aim of this study was to assess the reliability of the FFQ among a younger adult Peruvian population during the COVID-19 pandemic. Results suggest good reliability, measured as internal consistency of this FFQ (Cronbach’s alpha = 0.82) (32, 54). When assessing internal consistency within food groups, only three out of the fourteen food groups presented poor internal consistency (Cronbach’s alpha < 0.50) (31). Several reasons could explain the relatively lower internal consistency among these three food groups, including the smaller number of items in certain food groups (e.g., stews), and the high heterogeneous intake of food items within food groups (e.g., cereals). Future studies facing changes in their dietary intake data collection methods due to extenuating circumstances should aim at validating their instruments within the context where the study is conducted. Having valid dietary intake instruments can offer valuable insights for assessing and comparing dietary intakes across the population and between different time periods. Additionally, these instruments can provide valuable information about compliance with dietary guidelines, and examine the factors associated with a differential uptake of dietary recommendations at the country and international level using the most updated recommendations for ensuring a healthy and sustainable diet (55–57).

### Strengths and limitations

This is the first study, to our knowledge, to assess the validity and reliability of a FFQ applied during the COVID-19 pandemic in a LMIC. Despite the many difficulties in assessing dietary intakes during this period, including the impossibility of conducting face-to-face studies with trained fieldworkers, the YLS managed to adapt its methods to a varied population across different regions of Peru. However, as the FFQ was self-administrated *via* an online survey, there are several limitations to consider. First, online self-administration can challenge technologically unskilled and less educated participants (58). Although web-based users can be easily distracted, studies have shown that online surveys have a higher survey competition rate and are equally reliable compared with paper-based questionnaires (59, 60). Second, self-administered questionnaires reduce the interaction and communication between participant and interviewer, placing a cognitive burden depending on the questionnaire presentation (61). Our study aims at providing clear instructions and memory aids based on the suggestions from expert validation and previous face-to-face pilot, reducing the cognitive burden and respondent fatigue (5). In addition, the inclusion of photos of portion sizes and closed-ended frequency options contribute to reducing the cognitive burden but also minimizes coding time and transcription errors or misinterpretations that might not be able to clarify when responding to a self-administered survey (62). Third, BMI estimations relied on self-reported weight and height, introducing a potential bias due to the tendency for overreporting height and underreporting weight and BMI among individuals with overweight and obesity from different countries across the globe (63, 64). However, studies among younger adults have suggested that despite the relative overestimation of height and underestimation of weight, self-reported data is accurate for nutritional status classification based on BMI (65, 66). Fourth, shifting from interviewer-based to self-administered can affect the participant’s answering process due to the method’s impersonality and the lack of rapport and control over the order of the questionnaire (61). However, self-administered questionnaires have the advantage of offering a safer space for the disclosure of sensitive behaviors, reducing the risk of misreporting behaviors that are perceived as socially undesirable (67). Several health-related studies comparing interviewer-based and self-administered questionnaires have concluded no differences in data quality between these two administration modes (68–71). Few studies have compared differences in dietary intakes between interviewer-administered and a self-administered questionnaires and have reported different results. Similar findings were reported by a self-administered and an interviewer-administered 24 h recall (72), whereas higher EI misreporting among respondents of a web-based self-administered FFQ when compared to a trained interviewer-based FFQ (73), and small differences in the estimation of nutrients intake between a diet history recall and a self-administrated FFQ not affecting the prediction of disease outcomes (74) have been reported by different studies. Additionally, self-administered web-based FFQs have also proved to be valid when compared to food records and paper-based FFQs (75, 76). Further studies comparing different data collection methods, interviews, and assessment modes for measuring dietary intakes are needed to estimate the validity and reliability of cost-effective and time-saving instruments that can be used when facing mobility and resource restrictions such as the ones endured during the COVID-19 pandemic. Despite the limitations encountered, we believe this online self-administered FFQ was the most reliable option for assessing dietary intakes during the COVID-19 pandemic, offering a less-expensive and valid option for futures studies within the Peruvian population seeking to estimate diet-related outcomes. Based on our experience, reducing the number of food items, without affecting the food group’s internal consistency, as well as providing visual aids and clear instructions, can contribute to reducing the possible respondent fatigue and miscomprehension of frequency options behind the group of misreporters of EI.

## Conclusion

Our study is the first, to our knowledge, to assess the validity and reliability of an online self-administered FFQ among a younger-adult population in Peru during the COVID-19 pandemic. We describe the development process for elaborating this FFQ including the selection of food items, portion sizes, and food frequency response options. We classified 31% of the participants as misreporters, with a higher risk of O-R among men and the less educated, and a higher risk of U-R for women and the higher educated. These results are similar to previous studies assessing misreporting of EI, making our FFQ a valid instrument for assessing dietary intakes. Also, our questionnaire proved to have a good internal consistency based on Cronbach alpha, making it a reliable instrument for the context and population under study. Despite quantifying dietary intake as a complex task susceptible to inaccuracies (5), FFQs are still useful for informing dietary guidance and public health policy (12). Therefore, it is key to identify the characteristics associated with misreporting and take advantage of this information for mitigating these biases in future studies assessing dietary intakes across the population. Further research adapting dietary intake data collection methods should aim at validating their instruments to provide trustworthy information for public health researchers and policymakers targeting malnutrition.

## Data availability statement

The original contributions presented in this study are included in the article/Supplementary material, further inquiries can be directed to the corresponding authors.

## Ethics statement

The studies involving human participants were reviewed and approved by the Instituto de Investigación Nutricional, IIN (180-2002/CEI-IIN) and the Oxford Department of International Development (SSD/CUREC2/07-026). Approval for the data under analysis was obtained from the IIN ethical board in June 2021 (157-2021/CIEI-IIN). The patients/participants provided their written informed consent to participate in this study.

## Author contributions

KC-Q, AS, and KV designed the research. MV-S, KC-Q, KM-C, and AS designed the methodology for the research. AH-A and NL-B performed the statistical analysis. MV-S, KC-Q, AS, AH-A, and NL-B analyzed and interpreted the data. MV-S led and wrote the draft of the manuscript. KC-Q critically revised and edited the final draft of the manuscript. AH-A, KM-C, NL-B, LA, MF, MP, AS, and KV provided comments, revised the manuscript, and approved the final version. KC-Q and KV confirmed that they had full access to the data in the study and final responsibility for the decision to submit for publication. All authors contributed to the article and approved the submitted version.
