# Supplementary material for: Development of an online food frequency questionnaire and estimation of misreporting of energy intake during the COVID-19 pandemic among young adults in Peru
Source: Front Nutr. 2022 Aug 24;9:949330. doi: 10.3389/fnut.2022.949330 (PMC9449423; doi:10.3389/fnut.2022.949330)
Supplement: Supplementary file 1 [file Data_Sheet_1.pdf]

Development of an online food frequency questionnaire and estimation of misreporting of energy intake during the COVID-19 pandemic among young adults in Peru – Supplementary Material

Table 1. Cronbach Alpha for overall FFQ and food groups

All food categories	Alpha	Observations
Cronbach's Alpha Total	0.8205	
Cronbach's Alpha per Item		
Group 1: Cereals	0.814	494
Group 2: Whole grains	0.819	494
Group 3: Starchy	0.809	494
Group 4: Stew/Menestras	0.811	494
Group 5: Nuts and seeds	0.816	494
Group 6: Dairy	0.806	494
Group 7: Animal protein	0.803	494
Group 8: Seafood	0.807	494
Group 9: Vegetables	0.810	494
Group 10: Fruits	0.802	494
Group 11: Refined cereals	0.801	494
Group 12: Added fats	0.802	494
Group 13: Added sugars	0.810	494
Grupo14: Alcohol drinks	0.818	494

Table 2. Cronbach Alpha for food groups and food items

**Category 1: Cereals (1,2,3,8)**	**Alpha**	**Observations**
Cronbach's Alpha Total	0.339	
Cronbach's Alpha per Item		
Rice	0.211	491
Noodles	0.297	487
Oatmeal	0.371	463
Bread (any type)	0.219	471
**Category 2: Whole grains (4, 9, 10)**	**Alpha**	**Observations**
Cronbach's Alpha Total	0.539	
Cronbach's Alpha per Item		
Quinoa	0.593	377
Machika	0.333	153
Corn	0.480	430
**Category 3: Starchy (5, 6, 7, 11)**	**Alpha**	**Observations**
Cronbach's Alpha Total	0.500	
Cronbach's Alpha per Item		
Yucca	0.354	421
Sweet potato	0.360	430
Green/Ripe Banana	0.490	432
Potato	0.502	488
**Category 4: Lentils/Menestras (12, 13)**	**Alpha**	**Observations**
Cronbach's Alpha Total	0.270	
Cronbach's Alpha per Item		
Lentils, beans, or similars		481
Chocho or Tarwi		190
**Category 6: Dairy (22-26)**	**Alpha**	**Observations**
Cronbach's Alpha Total	0.644	
Cronbach's Alpha per Item		
Whole Milk	0.550	339
Fresh Milk	0.599	366
Yogurt	0.602	431
Fresh cheese	0.624	444
Buttery cheese	0.587	161
**Category 7: Animal protein (27-34)**	**Alpha**	**Observations**
Cronbach's Alpha Total	0.791	
Cronbach's Alpha per Item		
Chicken	0.780	488
Beef	0.767	431
Pork	0.762	414
Ham (all kinds)	0.757	271
Sausage, Bacon, or Cecina	0.758	245
Hot-Dog (all kinds)	0.764	300
Offal (liver, chicken gizzards, heart, others)	0.779	406
Egg	0.777	490
**Category 8: Seafood (35, 36, 37)**	**Alpha**	**Observations**
Cronbach's Alpha Total	0.609	
Cronbach's Alpha per Item		
Fish	0.476	458
Canned Tuna	0.512	392

Seafood	0.528	272
**Category 9: Vegetables (38, 39, 40, 41)**	**Alpha**	**Observations**
Cronbach's Alpha Total	0.603	
Cronbach's Alpha per Item		
Choclo corn	0.526	413
Fresh or Cooked Salads	0.578	475
Pumpkin locro (locro de zapallo)	0.527	341
Vegetables (stews and sauteed)	0.498	406
**Category 10: Fruits (42-47)**	**Alpha**	**Observations**
Cronbach's Alpha Total	0.749	
Cronbach's Alpha per Item		
Regular banana	0.737	468
Papaya, watermelon, pineapple, melon, grapes, others	0.669	465
Apple, pear, tangerine, orange, passion fruit, others	0.691	477
Dried plum and raisin	0.717	206
Avocado	0.728	429
Natural Fruit Juices	0.725	436
**Category 11: Refined cereals (48, 49, 50, 54, 55)**	**Alpha**	**Observations**
Cronbach's Alpha Total	0.814	
Cronbach's Alpha per Item		
Milk desserts	0.775	347
Milk-Free Mazamorras	0.760	334
Cakes/Cake	0.750	393
Flaked Cereals or Sweet Rings	0.790	245
Sweet Cookies	0.808	340
**Category 12: Added fats (15, 16, 17, 53, 56, 58-66)**	**Alpha**	**Observations**
Cronbach's Alpha Total	0.869	
Cronbach's Alpha per Item		
Margarine or Butter	0.866	320
Mayonnaise	0.862	388
Marmalade or Honey	0.867	342
Salty Snacks	0.862	289
Churros	0.859	135
Fritters At Home	0.864	481
Grilled chicken	0.856	441
Salchipapas	0.853	302
Broaster Chicken	0.855	332
Pizza	0.860	288
Tamale	0.866	311
Stuffed potato	0.858	266
Beef or Chicken Empanadas	0.856	224
Burger	0.857	282
**Category 13: Added sugars (18-21, 51, 51, 57, 71, 72)**	**Alpha**	**Observations**
Cronbach's Alpha Total	0.754	
Cronbach's Alpha per Item		
Soda	0.706	420
Artificial Nectars	0.710	319

Artificial Soft Drinks	0.706	268
Energy Drinks	0.712	226
Chocolate	0.738	379
Treats such as lollipop, hard or soft candy, others	0.733	284
Sugary drinks such as fruit drinks, chicha, emollient, infusions, coffee, quinoa, oatmeal, others	0.752	430
Sugar	0.767	449
**Category 14: Alcohol drinks (67-70)**	**Alpha**	**Observations**
Cronbach's Alpha Total	0.867	
Cronbach's Alpha per Item		
Wine	0.871	230
Beer	0.822	237
Pisco, Vodka, Rum	0.781	136
Pisco, Vodka, Rum with soda	0.841	178
